# The Broken *MLL* Gene Is Frequently Located Outside the Inherent Chromosome Territory in Human Lymphoid Cells Treated with DNA Topoisomerase II Poison Etoposide

**DOI:** 10.1371/journal.pone.0075871

**Published:** 2013-09-25

**Authors:** Sergey I. Glukhov, Mikhail A. Rubtsov, Daniil A. Alexeyevsky, Andrei V. Alexeevski, Sergey V. Razin, Olga V. Iarovaia

**Affiliations:** 1 Department of Molecular Biology, Faculty of Biology, Lomonosov Moscow State University, Moscow, Russia; 2 Institute of Gene Biology RAS, Moscow, Russia; 3 LIA 1066 French-Russian Joint Cancer Research Laboratory, Villejuif, France–Moscow, Russia; 4 A.N. Belozersky Institute for Physical and Chemical Biology, Lomonosov Moscow State University, Moscow, Russia; 5 Department of Bioengineering and Bioinformatics, Lomonosov Moscow State University, Moscow, Russia; 6 Scientific Research Institute for System Studies (NIISI RAN), Moscow, Russia; CCR, National Cancer Institute, NIH, United States of America

## Abstract

The mixed lineage leukaemia (MLL) gene is frequently rearranged in secondary leukaemias, in which it could fuse to a variety of different partners. Breakage in the MLL gene preferentially occurs within a ~8 kb region that possesses a strong DNA topoisomerase II cleavage site. It has been proposed that DNA topoisomerase II-mediated DNA cleavage within this and other regions triggers translocations that occur due to incorrect joining of broken DNA ends. To further clarify a possible mechanism for MLL rearrangements, we analysed the frequency of MLL cleavage in cells exposed to etoposide, a DNA topoisomerase II poison commonly used as an anticancer drug, and positioning of the broken 3’-end of the MLL gene in respect to inherent chromosomal territories. It was demonstrated that exposure of human Jurkat cells to etoposide resulted in frequent cleavage of MLL genes. Using MLL-specific break-apart probes we visualised cleaved MLL genes in ~17% of nuclei. Using confocal microscopy and 3D modelling, we demonstrated that in cells treated with etoposide and cultivated for 1 h under normal conditions, ~9% of the broken MLL alleles were present outside the chromosome 11 territory, whereas in both control cells and cells inspected immediately after etoposide treatment, virtually all MLL alleles were present within the chromosomal territory. The data are discussed in the framework of the “breakage first” model of juxtaposing translocation partners. We propose that in the course of repairing DNA topoisomerase II-mediated DNA lesions (removal of stalled DNA topoisomerase II complexes and non-homologous end joining), DNA ends acquire additional mobility, which allows the meeting and incorrect joining of translocation partners.

## Introduction

Chromosomal translocations are believed to cause different neoplasias, including leukaemias (for a review, see [1,2]). Translocations occur as a result of the incorrect repair of DNA double-stranded breaks (DSBs) by the non-homologous end joining (NHEJ) repair system [3-5]. Consequently, the introduction of DSBs increases the risk of translocations. DSBs are introduced to DNA directly by ionizing irradiation and by different genotoxic agents or as a result of errors in other DNA lesion repairs. Different genotoxic agents are widely used in anticancer therapy. Inhibitors of DNA topoisomerase II (topo II) constitute a large group of modern anticancer agents [6-10]. A variety of topo II-specific anticancer drugs inhibit the re-ligation step of the catalytic cycle of topo II. These drugs are known as topo II poisons [11]. The inability of topo II to re-ligate breaks introduced into DNA results in the accumulation of DSBs [12], with topo II remaining bound to the 5’ broken ends of DNA chains. In resting cells, the DSBs introduced by topo II can eventually be repaired [13,14], whereas in cycling cells, the inability to repair these lesions during the short period of time before mitosis can cause cell death. These facts explain the selective toxic effect of topo II poisons on fast-proliferating cancer cells. However, there are other proliferating cells in an adult organism, in particular lymphoid and erythroid cells. These cells are also preferentially affected by topo II-specific drugs, which could either cause cell death or stimulate the production of chromosomal translocations. As a result of these translocations, novel types of cancer cell clones could originate. Indeed, it has been shown that chemotherapy of solid tumours with DNA topoisomerase II poisons frequently results in the development of so-called secondary leukaemias [15-18]. This phenomenon has been studied extensively. The results obtained suggest that secondary leukaemias are associated with chromosomal translocations, which generate chimerical genes (for a review, see [19-21]). The spectrum of genes affected by translocations in secondary leukaemias is clearly non-random, which could be explained both by a certain specificity of initial translocation events and by the subsequent selection of cellular clones that have an advantage in survival and proliferation [22]. The mixed lineage leukaemia (MLL) gene is rearranged in approximately 33% of all studied cases of secondary leukaemias [23], where it can be found fused to many (> 70) different partners [24,25]. The majority of all known *MLL* rearrangements initiate within a 8.3 kb breakpoint cluster region (BCR) containing a strong *in vivo* DNA topoisomerase II cleavage site [26,27]. This suggests that preferential cleavage of the *MLL* gene by DNA topoisomerase II makes it a frequent partner in different translocation events.

It is worth noting that for a translocation to occur, a broken gene needs to meet another broken gene in a nuclear space. To fulfil this condition, the recombination partners must be located close to each other in the nuclear space either permanently or for a considerable period of time. The territorial organization of interphase chromosomes [28-30] creates an obstacle for potential translocation partners to meet. Indeed, chromosomal territories do not significantly overlap and the positions of genes within chromosomal territories appear to be restrained [31]. The apparent paradigm may have several explanations. It was reported that in spite of the above-described constraints, some genes from different chromosomes are located close to each other, for example, when these genes are recruited to the same transcription factory [32,33]. It is also worth nothing that the ends of broken DNA chains have been reported to acquire enhanced mobility [34]. Finally, the radial positions of genes involved in treatment-related chromosomal rearrangements appear to change in cells treated with topo II poisons [35,36].

To obtain more insight into the possible mechanism of chromosomal translocations underlying secondary leukaemias, we studied the localisation of the *MLL* gene in respect to a particular chromosomal territory and mutual localisation of *MLL* gene broken ends in human cultured lymphoid cells treated with a topo II poison etoposide commonly used in anticancer therapy [7]. Using 3D-FISH with break-apart probes, we observed spatially separated broken ends of the *MLL* gene in approximately 17% of cells. Furthermore, we found that after incubation of cells with etoposide, followed by a short recovery period, a significant portion of the *MLL* gene was located outside the chromosome 11 territory, whereas another gene located on the same chromosome, (*CCND1*), remained within the chromosomal territory. Taken together, these results suggested that peculiarities of the spatial organization of the *MLL* gene within a chromosomal territory contributed to the high level of participation of this gene in chromosomal translocations, originating as a result of the inhibition of DNA topoisomerase II by anticancer drugs.

## Results and Discussion

Poisoning of DNA topoisomerase II in human lymphoid cells by etoposide causes frequent breakage of the *MLL* gene followed by spatial separation of the broken ends.

**Figure 1:**
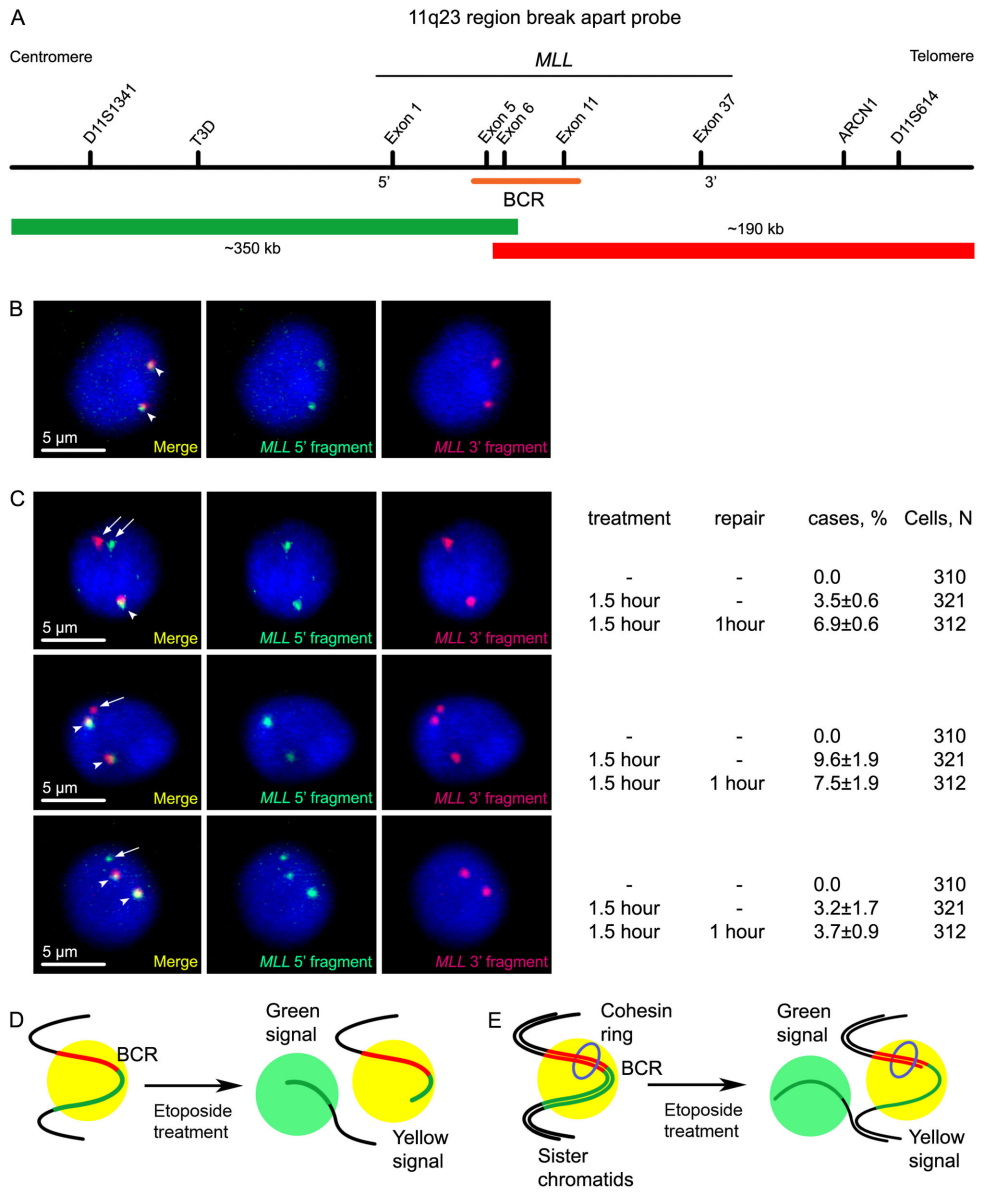
DNA breaks within the *MLL* gene breakpoint cluster region upon etoposide treatment. A) A map of the *MLL* locus with the positions of regions visualised by the LSI MLL break-apart hybridization probe that covers approximately 350 kb upstream of the BCR (green) and approximately 190 kb downstream of the BCR (red). B) Images of untreated Jurkat cells, DNA stained with DAPI (blue), genomic locus containing the 5’ fragment of the *MLL* gene (green) and the 3’ fragment of the *MLL* gene (red). Arrowheads show the double coloured spots. C) Different cases of staining of the *MLL* gene by the LSI MLL break-apart hybridization probe in Jurkat cells treated with etoposide: upper row – a cell with one double coloured spot (arrowhead), representing a non-cleaved *MLL* allele, and two single-coloured spots, representing the upstream and downstream ends of a broken *MLL* allele (arrows); middle and the lower rows - cells with two double coloured spots (arrowheads) and one additional red or green spot, respectively (arrow). The percentage of nuclei representing each of the shown patterns and number of the analysed cells are indicated to the right of section C. D,E) Schemes demonstrating the possible ways to generate the staining patterns are shown in section C.

Although it is known that treatment of Jurkat cells (cultured human lymphoid cells) with topo II poison etoposide causes cleavage of the *MLL* gene within a relatively short breakpoint cluster region [26,27], it was not clear whether the broken DNA ends are separated or remain in close proximity. In cells that have replicated their DNA, the broken ends of one chromatid could be held in close proximity due to cohesion of this chromatid with a non-damaged sister chromatid. Before DNA replication, broken DNA ends could be held together by the compact packaging of chromatin or due to an interaction between two subunits of DNA topoisomerase II bound to the opposite sides of a DSB. To analyse the behaviour of the broken ends of the *MLL* gene, we used a LSI MLL dual color, break apart rearrangement probe, commercially available from Vysis. This probe consists of a 350 kb portion centromeric to the *MLL* gene BCR labelled in SpectrumGreen and an approximately 190 kb portion largely telomeric to the BCR labelled in SpectrumOrange (Figure 1A). Consequently, the upstream part of the *MLL* gene was stained and detected as a green signal in microscopy images, whereas the downstream part of the *MLL* gene was detected as a red signal. The LSI MLL probe was used to visualise the *MLL* gene in control Jurkat cells, cells treated with etoposide (100 µg/ml, 1.5 h) and cells treated with etoposide and cultivated for 1 h in a culture medium without etoposide. FISH was performed under conditions allowing the subsequent analysis of the distribution of the signals in 3D space (3D-FISH). In nuclei bearing the normal allele of the *MLL* gene targeted by the LSI MLL probe, we would expect the signals of both fluorophores to co-localise to the same spot. In the nucleus of a diploid cell, two coloured spots with co-localising signals should be present (Figure 1B). In the case of a nucleus bearing an *MLL* allele broken at the BCR, we would expect one coloured spot with co-localising signals (normal allele) and two differently coloured spots representing the two separated parts of the broken *MLL* allele. Inspection of Jurkat cells stained with the LSI MLL probe after a 1.5 h incubation with etoposide demonstrated that in 3.4% of nuclei the *MLL* gene was broken at or close to the BCR and that the broken ends were separated in space (Figure 1C). This was concluded based on the fact that we observed three coloured spots (actually, stained regions in a 3D space): one with co-localised green and red colours, one red spot and one green spot. Importantly, in cells that were not treated with etoposide, only co-localised signals were detected. It is thus reasonable to assume that cleavage of the *MLL* gene, resulting in separation of the green and red signals, is mediated by DNA topoisomerase II. The percentage of cells bearing separated segments of the *MLL* gene increased (up to 7.7%) after the 1 h incubation of etoposide-treated cells in etoposide-free medium. This result suggested that the percentage of *MLL* alleles cleaved by topo II upon incubation of the cells with etoposide exceeded 3.4%; however, some of the cleavages were not visualised directly after treatment because the broken ends remained in close spatial proximity. It is possible that the processing of DNA topoisomerase II cleavable complexes or other steps in the repair process promote the spatial separation of the broken ends of the *MLL* gene.

In a number of cases (12.4% right after incubation with etoposide and 10.9% after 1 h cultivation of etoposide-treated cells in etoposide-free medium), we observed two co-localising signals and only one separate (either green or red) signal, as shown in Figure 1C (second and third row). These staining patterns could be explained in a couple of different ways. First, they could be generated by cleavage of the *MLL* gene at a considerable distance from the BCR, resulting in the splitting of either the green or red signal (Figure 1D). A part of the region stained by either the green or red constituent of the dual colour probe would remain linked to the region stained by a different colour (co-localising signals), whereas the other part could be spatially separated, thus giving rise to a single-colour signal (green or red). In this case, parts of a split coloured region would yield a less intensive signal than the non-split coloured region. Unfortunately, resolution of our 3D models was not sufficient enough to perform accurate estimations of the sizes of the coloured regions.

Second, some of the yellow spots shown in these figures may represent overlapping signals from two copies of the *MLL* gene located on sister chromatids. If a single chromatid is broken at the BCR of the *MLL* gene, it is possible that one end of the *MLL* gene could be spatially separated, whereas the other one could be retained in the complex due to a cohesion with its sister chromatid (Figure 1E). Distribution of cohesin in most of the genomic regions appears to be stochastic. Thus, a spatial separation of each of the MLL ends could be equally probable. This model is indirectly supported by an observation that the percentage of nuclei harbouring two mixed colour signals and one single colour (red or green) signal slightly decreased after 1 h cultivation of etoposide-treated cells in etoposide-free medium (Figure 1C). One would expect the repair process to proceed more efficiently in the presence of a sister chromatid due to the “freezing” of at least one end of the broken DNA chain or due to the involvement of a homologous repair pathway. However, in the framework of this model, it is difficult to explain why we have not seen a situation where both ends of the putative broken chromatid are spatially separated from a sister chromatid (i.e., two yellow signals and two single-colour (red and green) signals).

### Radial positions of intact and broken MLL alleles in cells treated with etoposide

**Figure 2:**
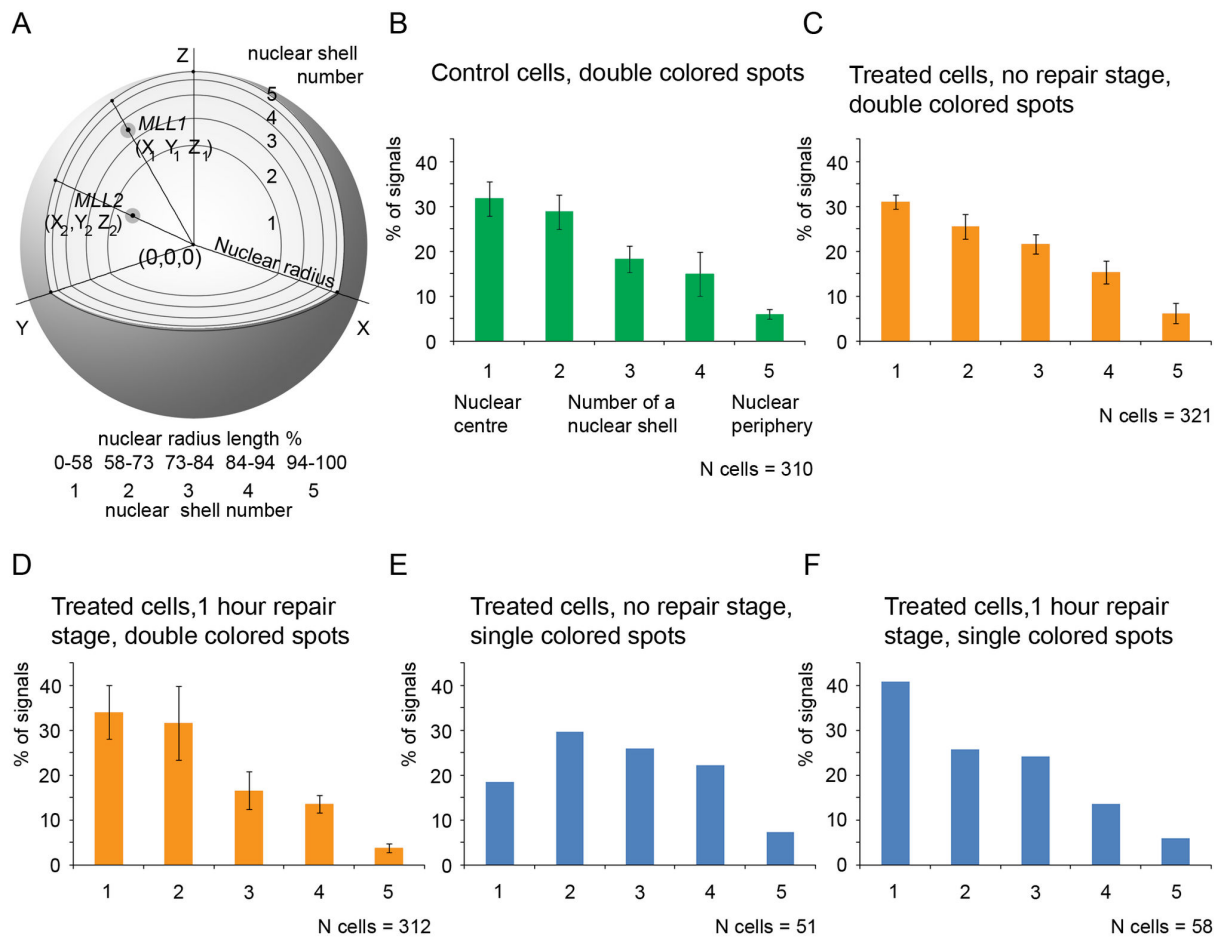
Principle of analysis of the radial positions of the *MLL* gene on 3D models. A) A schematic illustrating partitioning of the nucleus into five shells of equal volume. The nucleus is present as an ideal sphere within the scheme. The nuclear centre was assumed to have coordinates (0,0,0). The nuclear space was divided into five equal volumes – one central sphere and four peripheral shells. The borders of these shells are located at a distance of 58%, 73%, 84%, 94% and 100% of the nuclear radius length. The positions of the *MLL* gene (gray circles on the scheme) were documented in respect to the individual shells and nuclear centre. B-D) Distribution of double-colored signal between the central sphere and four peripheral shells in control cells (B), cells exposed to etoposide (C) and cells exposed to etoposide and then incubated for 1 h in etoposide-free medium (D). The average results of 3 independent experiments (more than 100 cells inspected in each experiment) are presented. The columns show mean value for three replicas, bars show the standard error. E,F) Distribution of broken MLL alleles (single-colored spots) in cells exposed to etoposide (E) and cells exposed to etoposide and then incubated for 1 h in etoposide-free medium (F). Because of the small number of broken alleles observed in each experiment, the data obtained in 3 independent experiments were combined.

It has been proposed to characterise the nuclear localisation of chromosomal territories and individual genes in terms of their so-called radial positions [28-30]. A radial position is the average distance between the centre of mass of the object under study (a gene or a chromosomal territory) and the centre of mass of the nucleus expressed as a percentage of the nuclear radius. Although the radial positions of genes and chromosomal territories within the nucleus of a given type of cell are constant, they could change under some specific conditions. In a previous study, we demonstrated that the radial position of the human *ETO* gene changed in cells exposed to etoposide [35]. Hence, we wondered if the same was true for the *MLL* gene or, more precisely, if the radial positions of the broken ends of the *MLL* gene differed from the radial positions of the non-damaged *MLL* gene. To this end, we divided the volume of all inspected nuclei (see the previous section) into 5 spherical shells of equal volume and documented the positions of the centres of the *MLL* signal in respect to these shells (Figure 2A). The results obtained are shown in Figure 2B-F. It is evident that radial positions of non-damaged (or broken but non-separated) *MLL* alleles are virtually the same in control cells (Figure 2B), cells incubated with etoposide (Figure 2C) and cells treated with etoposide and then incubated for 1 h in etoposide-free medium (Figure 2D). In contrast, positions of separated ends of broken *MLL* alleles showed a clear shift toward the nuclear periphery right after exposure of cells to etoposide (Figure 2E). The observed difference in distribution of broken and intact *MLL* alleles disappeared after incubation of the cells in etoposide-free medium for 1 h (Figure 1F). Although the functional significance of these positional changes, if any, is not clear, it appears that the spatial position of the *MLL* gene became less constrained as a result of topo II-mediated cleavage. This effect could be complicated as the mobility of both broken and intact *MLL* alleles could be affected by other DNA breaks, which are likely to occur within the same chromosomal territory as a result of etoposide treatment.

### In cells treated with etoposide, the downstream portion of the *MLL* gene is frequently located outside the chromosome 11 territory

**Figure 3:**
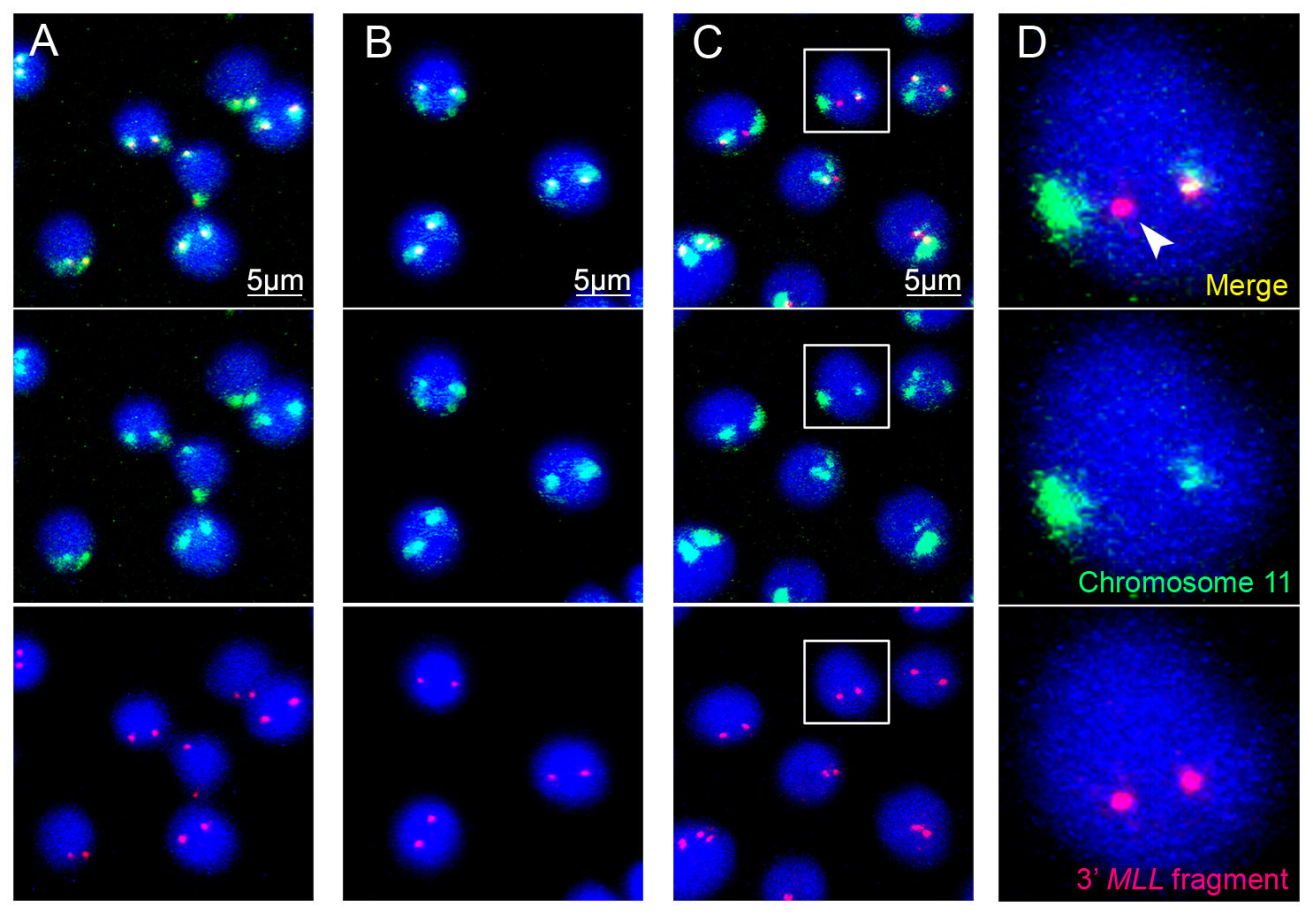
Relative positions of the *MLL* gene and chromosome 11 territory in control cells and cells treated with etoposide. Confocal images of cells made using the 3D-FISH technique; blue colour – DNA stained with DAPI, green colour – territory of chromosome 11, red colour - genomic locus containing the 3’ fragment of the *MLL* gene. A) Untreated cells. B) Cells treated with etoposide for 1.5 hour C, D) Cells treated with etoposide for 1.5 hour and cultivated for 1 h under normal conditions. The 3' fragment of the *MLL* gene thatmoves out of chromosome 11 territory is marked with an arrowhead. The enlarged nucleus indicated by the white square is shown in section D.

As outlined in the introduction, for a translocation to occur, the translocation partners located in different chromosomes need be brought in close proximity within the nuclear space. In this regard, it was of interest to analyse the position of the *MLL* gene in respect to the inherent chromosomal territory in normal and etoposide-treated cells. To this end, we stained the chromosome 11 territory with a commercially available probe conjugated with a modified FITC molecule and the *MLL* gene with the LSI MLL dual color, breakapart probe in the same cells. The disadvantage of this protocol was that both the chromosome territory and the 5’ end of the *MLL* gene were stained in green. For this reason, the 5’ end of the *MLL* gene was excluded from subsequent analyses. On the other hand, the 3’ end of the *MLL* gene (3’ *MLL*) was stained in red and was clearly visible both within and outside the chromosomal territory. Control cells, cells exposed to etoposide (100 µg/ml, 1.5 h) and cells exposed to etoposide and cultivated for 1 h under normal conditions were studied in parallel experiments. The results were documented and analysed using confocal microscopy and 3D modelling. Representative confocal sections are shown in Figure 3. In each case, more than 100 individual nuclei were examined, and each experiment was repeated three times. We found that in the nuclei of non-treated cells and following incubation of the cells with etoposide, the 3’ *MLL* was located within the territory of chromosome 11 (Figure 3A,B and Figure S1A,B). In contrast, in cells exposed to etoposide and incubated for 1 h under normal conditions, the 3’ *MLL* was frequently located outside the territory of chromosome 11 (Figure 3C,D and Figure S1C). Importantly, the 3’ *MLL* red signals present outside the chromosome 11 territory did not co-localise with the green signals. It is thus reasonable to suggest that they represent the 3’ end of a broken *MLL* gene. One may be surprised by the fact that we did not observe separate green signals (the other end of a broken *MLL* gene) outside the chromosome 11 territory. However, this could be explained by technical reasons, i.e., an inability to discriminate small green spots in the nuclei where larger chromosomal territories were stained in the same colour. The staining of chromosome territories is usually not uniform. Separate green spots were occasionally observed within the analysed nuclei. However, it is difficult to attribute these signals to the 5’ ends of the *MLL* gene as they could also represent an artefact produced by the imperfect staining of the chromosomal territories.

**Figure 4:**
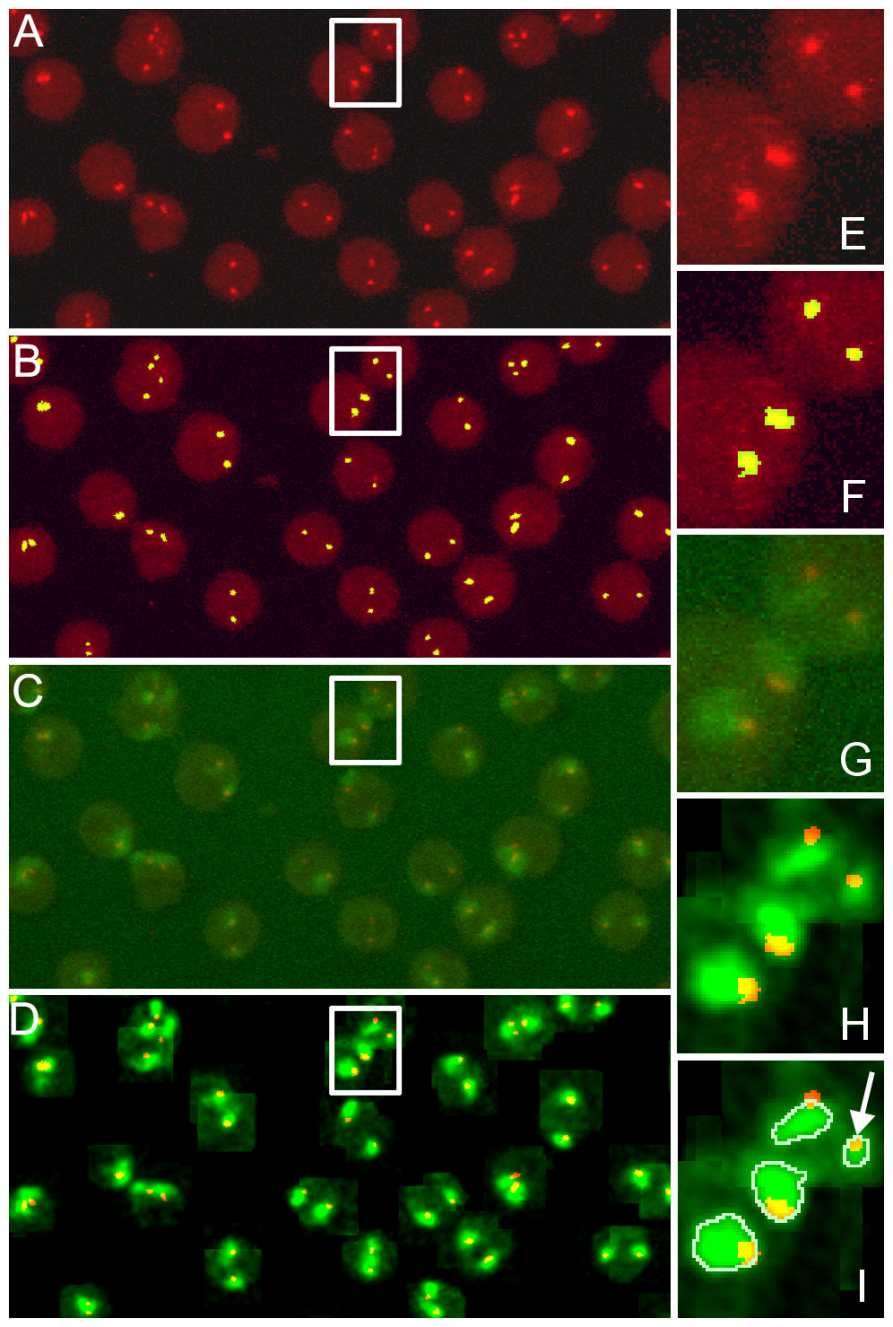
Verification of the *MLL* gene and chromosome 11 territory detection by a computer algorithm. Cells were treated with etoposide for 1.5 hour and cultivated under normal conditions for 1 h. In all cases, projections of the confocal sections are shown. A, B, E, F) Detection of the MLL signals. Original staining (red, A, E), detection of signals by the computer software (added green colour, resulting in yellow spots, B, F). C,D,G,H,I) Detection of a chromosome territory. Original staining (green, C, G), local brightness adjustment by the computer software (D,H), detection of the chromosomal territory by the computer software (I). Detected chromosome territories were automatically outlined in grey (I); during analysis border lines were not included in the territories. Chromosome territory rejected due to small size is indicated by an arrow (I).

To calculate the frequency of red signals outside chromosomal territories (escaping frequency), a special computational protocol was developed (see Materials and Methods). First, red signals were detected automatically in each confocal section. Visual control confirmed that more than 90% of all red signals for each slice of each image were detected correctly, with respect to human judgment (Figure 4A, B, E, F). The detection of chromosome territories was more complicated because in most cases it was not evident to an observer (expert) where exactly the borders of the chromosome territory should lie (Figure 4C, D, G, H, I). Clearly, computer programs would detect chromosome territories with a bit of uncertainty and the statistical data would be more relevant. On each image automatically detected chromosome territories were confirmed by visually comparing them to the initial images and detection parameters adjusted to make the results more adequate. The parameters for chromosome territory detection were the same for all images (due to good normalisation). In post processing extremely small (less than 100 pixels) or large (more than 500 pixels) chromosome territories were excluded to reduce the number of cases where normalisation failed and chromosome territories were detected incorrectly. The results of calculations made from three experiments three repeats each are presented in Table 1. As follows from these calculations, in control cells, virtually all *MLL* alleles resided within the territory of chromosome 11 (escaping frequency ~0.5% of 2128 signals analysed). Immediately after exposure to etoposide the escaping frequency of *MLL* alleles shows only small, although statistically significant (one-tailed Fisher’s exact test, p=0.04) increase (~1% of escaped alleles of 1190 analysed). In contrast, in cells treated with etoposide and cultivated for 1 h under normal conditions, ~9% of the MLL alleles were found outside the chromosome 11 territory. The difference with control cells was highly significant (one-tailed Fisher’s exact test, p<0.0001).

**Table 1:** Percentage of *MLL* and *CCND1* alleles escaping the territory of chromosome 11.

Series	Number of repeats	Fields of vision	Spots (total)	Escaping spots, number and % of total	Escaping spots (%), average of 3 repeated experiments and SE	P-value (exp vs control)
CCND1, 1 hour repair stage	3	12	1256	27 (2.1%)	2.7%±1.6%	0.28
CCND1, control cells	3	9	1042	18 (1.7%)	1.5%±0.8%	
*MLL*, no repair stage	3	11	1190	11 (0.9%)	0.9%±0.5%	0.04
*MLL*, 1 hour repair stage	3	12	1372	121 (8.8%)	7.2%±3.0%	<0.0001
MLL, control cells	6	19	2128	8 (0.4%)	0.5%±0.2%	

The column “Series” shows the analysed gene and conditions of treatment and repair stages. The column “Number of repeats” shows the numbers of independent pairs of experimental and control procedures. The column “Fields of vision” shows the number of images taken by computer analysis. The column “Spots (total)” shows the number of target gene alleles detected using the computer algorithm. The percentage of escaping spots was calculated either based on total figures (column “Escaping spots, number and % of total” or as an average of data obtained in 3 independent experiments (column “Escaping spots (%) , average of 3 repeated experiments and SE”). For eligibility criteria see “Materials and methods”. The column “P-value” shows the reliability of the differences between the control and experimental data; for “Series” and “*MLL*, 1 hour repair stage”, the p-value was calculated between three incorporated experiments and six incorporated controls. The line "*CCND1*, 1 hour repair stage" shows data about the relative positioning of the *CCND1* gene and chromosome 11 territory; experimental cells were treated for 1.5 hour and were incubated for 1 hour without etoposide. The line “*MLL*, no repair stage” shows data about the relative positioning of the *MLL* gene and chromosome 11 territory; experimental cells were treated for 1.5 hour. The line “*MLL*, 1 hour repair stage” shows data about the relative positioning of the *MLL* gene and chromosome 11 territory; experimental cells were treated for 1.5 hour and were incubated for 1 hour without etoposide.

To better understand the possible relationship between the observed mobility of the *MLL* gene cleaved by DNA topoisomerase II and the preferential participation of this gene in treatment-related chromosomal translocations, it was important to find out whether other genes located in the same chromosome (chromosome 11) demonstrate the same behaviour. To this end, we analysed the position of the cyclin D1 (*CCND1*) gene in respect to the chromosome 11 territory in normal and etoposide-treated cells. This gene was chosen because it is constitutively transcribed in Jurkat cells and does not participate in treatment-related translocations. We found that 1.7% of *CCND1* alleles were located outside the chromosome 11 territory already in non-treated cells. This figure virtually did not change after cell exposure to etoposide and subsequent incubation for 1h in etoposide-free medium (escaping frequency 2.1% of 1256 treated and 1.7% of 1042 untreated cells; one-tailed Fisher’s exact test, p=0.28). Hence, the preferential participation of the *MLL* gene in treatment-related translocations could be partially explained by the fact that upon DNA topoisomerase II-mediated cleavage of this gene the broken ends acquire increased mobility, which frequently results in the positioning of at least the downstream portion of the *MLL* gene outside the chromosome 11 territory.

### Concluding remarks

It is known that the *MLL* gene is frequently rearranged in treatment-related leukaemias. The presence of the so-called *in vivo* DNA topoisomerase II cleavage sites within the *MLL* BCR [26,27] suggests that the *MLL* gene could be preferentially cleaved by DNA topoisomerase II under conditions of treatment with topo II poisons. Subsequent incorrect repair of the introduced DSB(s) could result in chromosomal translocations [19,33]. Our results strongly corroborate this model. First, in agreement with the previously published data [33], we have demonstrated that cleavage of *MLL* within or close to the BCR region occurs quite frequently in cells exposed to etoposide, a topo II poison commonly used for anticancer therapy. Furthermore, we have demonstrated that in a short period of time following etoposide treatment of Jurkat cells, the downstream portion of the broken *MLL* gene frequently emerged outside the chromosomal territory. Taken as a whole, these findings argue that the structural organization of the *MLL* gene (presence of topo II interaction/cleavage sites) and its spatial organization within a chromosomal territory, allowing for its relocation outside the territory, may contribute to the frequent involvement of the *MLL* gene in treatment-related translocations. It is clear that, for translocations to occur, the ends of two broken chromosomes need to be located in close proximity. However, the mechanism for the juxtaposition of the broken ends of two different chromosomes remains obscure. Two models are discussed in the literature. The “contact first” model postulates that translocation partners are located close to each other in the nuclear space [37,38], whereas the “breakage first” model postulates that the broken ends of chromosomes are juxtaposed only after breakage [39]. Both models are supported by experimental data. Some authors found that DSB are positionally stable [40-43]. These findings corroborate the supposition that initial spatial proximity of DNA breaks determines the specificity of translocations. In other studies a long-range movement of broken DNA ends postulated by “breakage first” model was observed [34,39]. The results presented in this paper corroborate the “breakage first” model for *MLL*-involving translocation events. It appears that the enhanced mobility of the broken end(s) of the *MLL* gene facilitates contacts with different translocation partners. It has previously been reported that the broken DNA ends, produced under conditions of topo II poisoning, possessed increased mobility within the nucleus [34]. Our data argue that this increased mobility is not a direct consequence of DNA cleavage, but rather is related to the assembly of repair complexes. If this were not true, it would be difficult to explain the fact that increased mobility of the broken DNA ends was observed only after a 1 h incubation of etoposide-treated cells under normal conditions.

## Materials and Methods

### Cell culture

The Jurkat human lymphoid cell line was obtained from the collection of the Institute of Medical Genetics RAMS. Cells were grown in RPMI 1640 medium supplemented with 10% FBS at 37°C in a 5% CO_2_ incubator. Treatment of cells with etoposide (100 µg/ml) was performed in the same medium for 1.5 h.

### Preparation of cells for microscopy

Cells were attached to slides using Cell-Tak™ (BD Bioscience) according to the manufacturer’s protocol. The slides were incubated for 1 min in 0.3x PBS, fixed in 4% paraformaldehyde in 0.3x PBS (pH 7.4) for 10 min at room temperature and washed in 1x PBS. Cells were permeabilised at room temperature in 0.5% (w/v) Triton X-100 in 1x PBS for 10 min and incubated for 12 hour in 20% (v/v) glycerol/1x PBS at +4°C. Slides were frozen four times in liquid nitrogen; between the freezing stages, the slides were placed in 20% (v/v) glycerol/1x PBS. After the freezing step, the slides were incubated in 0.1 N HCl for 20 min at RT, washed in 1x PBS, treated with RNAse A (200 µg/ml) in 2x SSC for 30 min at 37°C, washed with 2x SSC and equilibrated in 50% (v/v) deionised formamide in 2x SSC for at least one week at +4°C.

### Visualisation of the *MLL* gene and chromosome 11 territory via 3D fluorescence in situ hybridization (3D-FISH)

We used the Vysis® LSI® MLL dual color, break apart rearrangement probe (Abbott) labelled with SpectrumGreen and SpectrumOrange fluorescent markers and the Aquarius® Whole Chromosome 11 Painting probe (CytoCell) labelled with a green fluorophore possessing the same characteristics as FITC. Genomic DNA from the cells on microscope slides was denatured in 70% (v/v) deionised formamide in 2х SSC for 15 min at 75°C. We used two types of probes for hybridization: (1) to find breaks in the *MLL* gene, the mixture contained 0.5 µl of Vysis® LSI® MLL dual color, break apart rearrangement probe (Abbott), 3.5 µl of Vysis® hybridization buffer and 1 µl of deionised water per slide; (2) to analyse the relative position of the *MLL* gene in respect to the chromosome 11 territory, the mixture contained 0.5 µl of Vysis® LSI® MLL dual color, break apart rearrangement probe (Abbott), 2.5 µl of Vysis® hybridization buffer, 1 µl of Aquarius® Whole 11 Chromosome Painting probe and 1 µl of deionised water per slide. The mixtures were incubated for 5 min at 75°C, following which they were applied to the preheated slides and mounted with rubber cement. Hybridization was carried out for 48 hours at 37°C in a humid chamber. Slides hybridised only with Vysis® LSI® MLL dual color, break apart rearrangement probe were washed three times for 10 min in 50% (v/v) formamide in 2x SSC at 52°C, for 10 min in 0.2x SSC at 52°C, for 5 min in 0.1% (v/v) NP-40 in 2x SSC at 52°C and for 3 min in 2x SSC at room temperature. Slides hybridised with both Vysis® LSI® MLL dual color, break apart rearrangement probe and Whole Chromosome Painting probe were washed for 2 min in 0.4x SSC-0.3% NP-40 at 72°C, for 1 min in 2x SSC-0.1% NP-40 at 72° С and 3 min in 2x SSC at room temperature. In all cases, the DNA was stained with DAPI (Invitrogen). For microscopy, the slides were mounted using mounting medium (Dako) suitable for fluorescent microscopy.

### Confocal microscopy

Images were obtained using the confocal laser scanning microscope Zeiss LSM 510 META with objective Plan-Apochromat 63x/1.40 oil. Lasers with wavelengths of 405 nm, 488 nm and 543 nm were used for excitation of the fluorescent markers. A typical confocal series contained from 12 to 22 slices. The top and bottom slices were excluded from the following analysis because of poor quality.

### Count of the broken MLL alleles’ number

*MLL* alleles were detected using Nemo software [44]. The *MLL* alleles and whole nuclei were detected as 3-dimensional objects. Parameters of the program were optimised for the best accordance between the original images of nuclei and genes and the result of the computer recognition. The population of analysed control cells, cells treated with etoposide and cells treated with etoposide and incubated without it for 1 hour contained 310, 321 and 312 cells correspondingly. Each population was consisted of samples obtained in 3 independent experiments (~100 cells taken for analysis from each experiment).

### *MLL* radial distribution building and analysis

Radial position of each particular allele were measured as a quotient of the distance between the centres of the allele and corresponded nucleus and the linear segment that begins in the nucleus centre passes through the allele centre and ends at the point of crossing with the nuclear surface. All distances were obtained using Nemo software [44]. The distribution of radial positions between five concentrically placed nuclear shells was counted. All the shells were of equal volume. Borders of the shells were located at 0%, 58%,73%,84%, 94%, 100% of nuclear radius length. These values accords to the borders of five concentrically placed shells of equal volume for a sphere. The number of analysed cells was the same as at the previous chapter of the "Material and Methods" paragraph.

### Analysis of a relative position of the *MLL* gene with respect to chromosome 11 territory

The 3’ fragment of the *MLL* gene was labelled with SpectrumOrange marker (detected as a red signal). The chromosome 11 territory was labelled with a fluorescent marker possessing the same characteristics as FITC (detected as a green signal). Nine series of experiments were carried out. Each series included confocal microscope images of control and etoposide-treated cells, several fields of view in each case. Further steps were applied separately to each image consisting of numerous slices, which were documented as separate graphical files (Table 1). Images from both control and etoposide-treated cells underwent the same protocol. The protocol is based on scripts written by authors in the *bash* and *Python* programming languages with the use of the *Python Imaging Library* and *Numpy* packages. The main steps were visually controlled. If necessary, the parameters of the programs were adjusted separately for each image to obtain the most adequate results.

#### Detection of *3’ MLL*

Pixels with values in the red channel above the threshold **r** were detected as significant. Groups of adjacent (in 3 dimensions: x, y, slice number) significant pixels were called spots. Spots smaller than 16 pixels were assumed to be noise and removed. The value of **r** was adjusted for each image by visualisation of the detected signals overlaid on source images, (Figure 4A,B). The possible values of **r** were diverse, spanning between 25 and 160.

#### Normalisation of the green channel

Gaussian blurring with σ=2 was applied to the green channel for all slices. For each detected spot, for each slice it spanned, a square neighbourhood of 50x50 pixels was constructed. For every such neighbourhood, the same normalisation procedure for green pixel values was applied: all values below the lower threshold (these were purported to be noise) were set to 0, all values above the upper threshold were set to 255 (these were purported to be signals that were not interesting in this case) and all values between them were stretched linearly. For every such neighbourhood, the lower threshold value was obtained as 0.3 quantile of possible pixel values in the green channel, whereas the upper threshold value was obtained as 0.99 quantile.

#### Detection of chromosome territories

For each neighbourhood of a spot (same as above), pixels with values in the green channel above threshold t = 180 were categorised as chromosome territories. For each image, visual control was performed (Figure 4C,D,G,H,I).

#### Computation of a spot escaping from the chromosome territory

Each spot was extended by one pixel in the x and y coordinates before measuring its escape. Escaping is defined as the percentage of pixels of a spot that do not belong to the chromosome territory. If escaping of a spot exceeds 80%, then the spot is considered to have escaped its chromosome territory. For each spot, its escape was calculated and saved in an Microsoft Excel file. Filter was applied to remove spots with either: red signal size out of range 16-150 pixels, chromosome territory within 20x20 box around red signal occupying less than 100 or more than 500 pixels. For each series, for control and etoposide-treated cells separately, the number of spots that escaped their territories and the number of spots that did not escape were calculated and saved as 2x2 tables. For every such table, decisions on the differences between the experimental and control set were made using the one-tailed Fisher’s exact test.

## Supporting Information

Figure S1 **Relative positions of the *MLL* gene and chromosome 11 territory in control cells and cells treated with etoposide.** Confocal images of cells made using the 3D-FISH technique; blue colour – DNA stained with DAPI, green colour – territory of chromosome 11, red colour - genomic locus containing the 3’ fragment of the *MLL* gene. A) Untreated cells. B) Cells treated with etoposide for 1.5 hour. C) Cells treated with etoposide for 1.5 hour and cultivated for 1 h under normal conditions. The 3' fragments of the *MLL* gene that are located outside of chromosome 11 territory are marked with arrowheads.
(TIF)
