# Supplementary figures and images for: The Broken MLL Gene Is Frequently Located Outside the Inherent Chromosome Territory in Human Lymphoid Cells Treated with DNA Topoisomerase II Poison Etoposide
Source: PLoS One. 2013 Sep 25;8(9):e75871. doi: 10.1371/journal.pone.0075871 (PMC3783379; doi:10.1371/journal.pone.0075871)

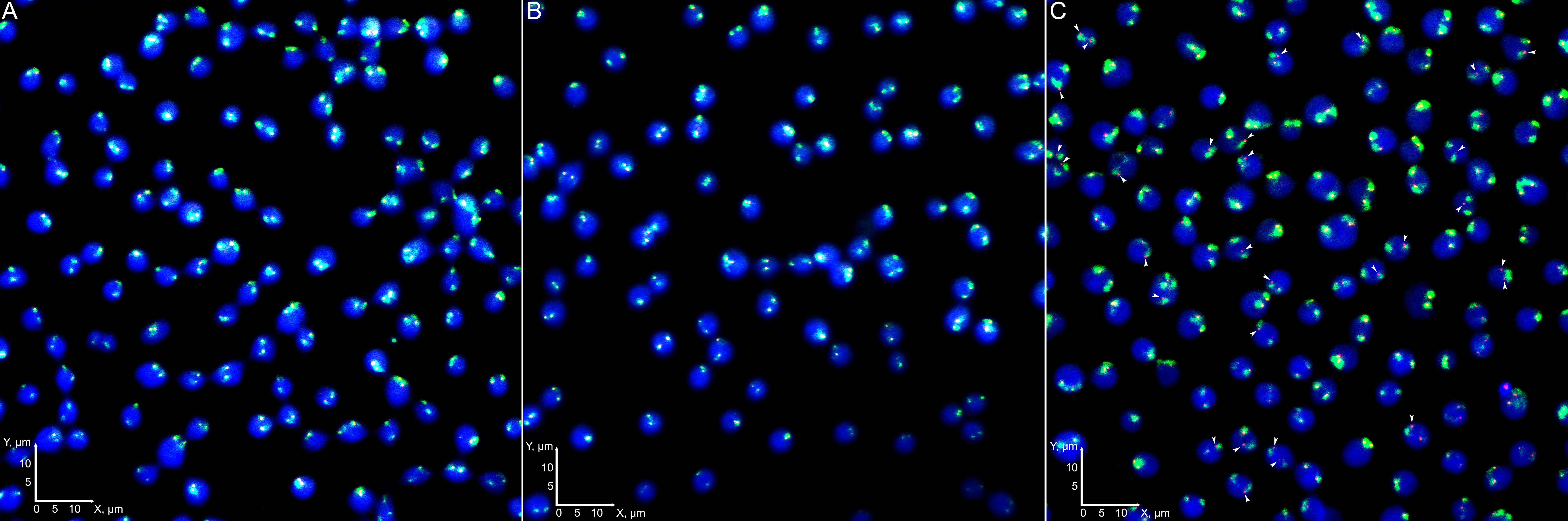

Supplement: Figure S1 — Relative positions of the MLL gene and chromosome 11 territory in control cells and cells treated with etoposide. Confocal images of cells made using the 3D-FISH technique; blue colour – DNA stained with DAPI, green colour – territory of chromosome 11, red colour - genomic locus containing the 3’ fragment of the MLL gene. A) Untreated cells. B) Cells treated with etoposide for 1.5 hour. C) Cells treated with etoposide for 1.5 hour and cultivated for 1 h under normal conditions. The 3' fragments of the MLL gene that are located outside of chromosome 11 territory are marked with arrowheads. (TIF) [file pone.0075871.s001.tif]
